# The effect of diabetes mellitus on outcomes of patients with nosocomial pneumonia caused by methicillin-resistant *Staphylococcus aureus*: data from a prospective double-blind clinical trial comparing treatment with linezolid versus vancomycin

**DOI:** 10.1186/s12879-016-1779-5

**Published:** 2016-09-06

**Authors:** Ozlem Equils, Christopher da Costa, Michele Wible, Benjamin A. Lipsky

**Affiliations:** 1 Pfizer Inc., Collegeville, PA USA; 2 University of Oxford, Oxford, UK; 3 University of Washington, Seattle, WA USA

**Keywords:** Linezolid, Diabetes mellitus, MRSA, Vancomycin, Pneumonia, Mortality, Outcome, Staphylococcus, Infection, Prognosis

## Abstract

**Background:**

The presence of diabetes mellitus increases the risk of several severe infections, but data on its effect on treatment outcomes in patients with nosocomial pneumonia (NP) caused by methicillin-resistant *Staphylococcus aureus* (MRSA) are limited.

**Methods:**

We retrospectively analyzed data from a double-blind, randomized, multi-center, international clinical trial of culture-confirmed MRSA NP that compared treatment with linezolid to vancomycin. Specifically, we evaluated the clinical and microbiologic outcomes of patients with and without diabetes in the modified intent to treat population at end-of-treatment (EOT) and end-of-study (EOS, 7–30 days post-EOT).

**Results:**

Among 448 enrolled patients 183 (40.8 %) had diabetes mellitus, 87 (47.5 %) of whom received linezolid and 96 (52.5 %) vancomycin. Baseline demographic and clinical characteristics were similar for the two treatment groups. Clinical success rates at EOS were 57.6 % with linezolid and 39.3 % with vancomycin, while microbiological success rates were 58.9 % with linezolid and 41.1 % with vancomycin. Among diabetic patients, rates of mortality and study drug-related adverse effects were similar between the treatment groups. Overall day 28 mortality rates were higher among diabetic patients compared to non-diabetic patients (23.5 vs 14.7 %, respectively: RD = 8.8 %, 95 % CI [1.4, 16.3]).

**Conclusions:**

Among diabetic patients with MRSA NP, treatment with linezolid, compared to vancomycin, was associated with higher clinical and microbiologic success rates, and comparable adverse event rates.

**Trial registration:**

NCT00084266.

**Electronic supplementary material:**

The online version of this article (doi:10.1186/s12879-016-1779-5) contains supplementary material, which is available to authorized users.

## Background

Persons with diabetes mellitus, compared with non-diabetic persons, have higher rates of impaired immunity [1, 2], decreased lung function [3, 4], and an increased risk for various types of infection, including pneumonia [5, 6]. Methicillin-resistant *Staphylococcus aureus* (MRSA) has emerged over the last decade as a common etiologic agent of nosocomial pneumonia, especially in intensive care units (ICUs). For example, the prevalence of MRSA as a cause of nosocomial pneumonia (NP) in intensive care units (ICU) has been reported as 37 % in Germany, 54 % in the US and 78 % in Asia and Latin America [5]. Patients with diabetes appear to be at increased risk for acquiring *S. aureus* pneumonia [6–8], and patients requiring renal dialysis are at risk for hospital-acquired pneumonia, healthcare associated pneumonia and ventilator associated pneumonia caused by multi-drug resistant pathogens [8]. A recent study by Haque and colleagues found that 28-day mortality rates were higher among ICU patients with MRSA pneumonia when they had comorbid diabetes [9].

Current guidelines for the management of adults with hospital-acquired, ventilator-associated and healthcare-associated pneumonia issued jointly by the American Thoracic Society and the Infectious Diseases Society of America (IDSA) recommend either linezolid or vancomycin as appropriate antibiotic agents for the treatment of MRSA nosocomial pneumonia (NP) [8]. The guidelines do not, however, address the potential for worse outcomes of this infection in a patient with diabetes, nor how the presence of this comorbidity might affect selection of antibiotic therapy. Presumably, this is based on a lack of published data on these issues.

We hypothesized that the immune and lung dysfunction commonly associated with diabetes may lead to a worse response to antibiotic treatment for MRSA pneumonia among these patients compared with non-diabetic patients. Further, because there has been a consistent trend in published trials toward better outcomes with treatment of MRSA pneumonia with linezolid than with other antibiotic agents [10–12] we wished to investigate the potential effect of underlying diabetes on treatment outcomes with linezolid versus vancomycin. To evaluate our hypothesis we conducted a post-hoc, subgroup analysis of data taken from a recent large, multicenter, prospective, randomized controlled study to examine the effect of diabetes mellitus on clinical and microbiologic outcomes in patients treated with either linezolid or vancomycin for culture-confirmed MRSA NP [12].

## Methods

We retrospectively analyzed data from a prospective, multicenter, international study comparing linezolid (600 mg twice a day) to vancomycin (15 mg/kg twice a day, with dose adjustment as necessary based on trough levels and creatinine clearance) administered for 7–14 days for the treatment of NP confirmed by culture to be caused by MRSA. The previously published main study provides details of the study design and overall results [12]. The study was approved by an institutional review board or ethics committee at each investigational site. Investigators obtained written informed consent from each patient or legally authorized representative. Briefly, male and female patients ≥18 years old were enrolled if they had NP (defined as at least two clinical signs and symptoms consistent with pneumonia [i.e., new or worsening cough or purulent sputum, auscultatory findings of pneumonia, dyspnea, tachypnea, hypoxemia or new respiratory failure requiring mechanical ventilation] acquired after 48 h in an inpatient healthcare or chronic-care facility, or after recent hospitalization [within 90 days] or undergoing chronic dialysis within 30 days), and a respiratory specimen for culture. Potential patients were not excluded based on their baseline blood glucose levels; no baseline hemoglobin A1c levels were recorded. Patients could receive up to two days of non-study antibiotic therapy prior to randomization or study entry.

A local, unblinded pharmacist prepared the study medication, monitored and adjusted vancomycin doses according to local protocols and measurements of trough vancomycin levels and renal function. The investigators and patients were blinded throughout the study. Pending culture results, all patients could initially receive an antibiotic with activity against aerobic gram-negative bacterial pathogens, but not one with activity against MRSA; mixed infections were treated with additional agents for gram-negative antibiotic coverage. Patients were clinically assessed at baseline, on day 3 and every 3 days during treatment. Respiratory cultures were repeated 48–72 h after treatment initiation, at end of treatment (EOT), and at end of study (EOS, defined as 7–30 days after EOT).

The current analysis included only the modified-intent-to-treat (MITT) population, defined as patients who received at least one dose of study drug and had culture-proven MRSA pneumonia. Since we used previously collected data without personal identifiers for this secondary analysis we did not obtain a separate ethics consent. We classified patients into those with, and those without, diabetes mellitus, based on the data recorded by the investigator on the comorbidity case report forms provided for each enrolled patient. Clinical outcome was primarily assessed by the investigator within 5 days of EOT and at EOS, with occasional override by the sponsor based on the criteria of Additional file 1: Table S1. Any revisions in classification of outcome were made before unblinding. Microbiologic responses were determined at EOT and EOS, based on the results of repeat cultures obtained from the original infection site. Additional file 1: Table S1 provides the definitions of clinical and microbiologic outcomes. We also assessed all treatment-related adverse events (AE), serious adverse events (SAEs), AEs that led to study drug discontinuation, and day 28 and day 60 (all-cause) mortality.

### Statistical analysis

To assess statistical differences in the distribution of baseline characteristics between the two treatment groups, we used one-way analysis of variance for continuous variables and Fisher’s exact test or the chi-square test, as appropriate, for categorical variables. Prior to analysis of clinical and microbiologic response and mortality, a Breslow-Day test was conducted to evaluate the homogeneity of treatment for the non-randomized diabetes subgroups. We excluded patients who were classified in the “unknown” (clinical) or “indeterminate” (microbiologic) categories from statistical analysis of response. To assess the association of variables with clinical response and mortality among patients with diabetes and MRSA NP, we performed multivariate logistic regression analyses. Prior to model building we took into consideration covariate reduction techniques: near zero variance, missingness and covariate correlations, associations and clusters. We identified variables for inclusion in the final multivariate logistic regression model based on selection in at least 50 % of 1000 bootstrap samples, where the model for each bootstrap was selected by backward elimination with an alpha stay criterion of 0.05. Baseline variables in the multivariate analysis included: type of antibiotic treatment; age; weight; sex; race; presence of infiltrate on chest x-ray; presence of pleural effusion; Acute Physiology and Chronic Health Evaluation (APACHE) score at baseline; presence of bacteremia; ICU admission at baseline; pathogen type (cultures positive for MRSA alone or in conjunction with other gram-positive, gram-negative or anaerobic organism[s]); type of ward or service on which the patient was hospitalized (surgical, medical, vs trauma); admission from a long term care facility; presence of co-morbidities (e.g., neoplastic, renal/urinary, pulmonary, hepatobiliary, vascular, gastrointestinal); medications at baseline; a history of undergoing renal dialysis; baseline blood glucose; smoking status (current, ex-smoker or nonsmoker); pneumonia type (ventilator-associated, hospital-associated or healthcare associated); and, minimum inhibitory concentration (MIC) of the organism against linezolid and vancomycin. We used 2-sided tests for all statistical comparisons and considered *p*-values <0.05 as statistically significant. For statistical procedures we used SAS, version 9.2 (SAS Institute, Cary, NC).

## Results

### Study population and patient demographics

Overall, 448 enrolled patients met the study entry criteria, 183 of whom had diabetes and 265 were non-diabetic. Among the patients with diabetes, 87 (47.5 %) were randomized to receive linezolid and 96 (52.5 %) to receive vancomycin. Among non-diabetic patients 137 (51.7 %) received linezolid and 128 (48.3 %) received vancomycin. The majority of patients with diabetes were insulin-treated at enrollment: 66.7 % among the linezolid-treated and 77.1 % among the vancomycin-treated patients.

Demographic and baseline characteristics were similar between the linezolid and vancomycin treatment groups (Table 1). Compared to the non-diabetic patients, the diabetic patients had a significantly higher percent: older than 50 years of age; heavier than 75 kg; with an APACHE II score ≥20; or, with a cardiac, vascular, renal or gastrointestinal comorbidity (Table 1). The diabetic and non-diabetic groups were not statistically different in their baseline microbiological results, vancomycin MICs, clinical pulmonary infection scores, percent of patients who were ventilated or had bacteremia, frequency of pleural effusion (43.7 vs 45.7 %, *p* = 0.70) or bilateral lung involvement on chest X-ray (72.1 vs. 66.4 % *p* = 0.17).

**Table 1:** Demographic and clinical characteristics of diabetic and non-diabetic patients treated with either linezolid or vancomycin for MRSA-nosocomial pneumonia in the modified intent to treat (MITT) population

	Diabetic Patients (*N* = 183)		Non-diabetic Patients (*N* = 265)	
Characteristic	Linezolid *N* = 87	Vancomycin *N* = 96	Linezolid *N* = 137	Vancomycin *N* = 128
Age (y), mean (SD) ***	67.1 (13.0)	69.8 (12.8)	58.2 (19.8)	56.1 (19.3)
Male Sex, *n* (%)	57 (65.5)	55 (57.3)	94 (68.6)	88 (68.8)
Race, *n* (%)				
White	59 (67.8)	60 (62.5)	97 (70.8)	92 (71.9)
Black	11 (12.6)	15 (15.6)	14 (10.2)	16 (12.5)
Asian	12 (13.8)	17 (17.7)	20 (14.6)	16 (12.5)
Other	5 (5.7)	4 (4.2)	6 (4.4)	4 (3.1)
Treatment duration (days), mean (SD)	9.7 (4.1)	9.1 (4.9)	10.1 (3.8)	10.0 (4.1)
Baseline blood glucose (mg/dl), n^a^/mean (SD)	83/176.3 (116.7)	91/167.8 (123.5)	129/164.7 (152.7)	121/174.9 (158.9)
Bacteremia, *n* (%)				
Yes	6 (6.9)	10 (10.4)	9 (6.6)	14 (10.9)
No	78 (89.7)	85 (88.5)	116 (84.7)	110 (85.9)
Unknown	3 (3.4)	1 (1.0)	12 (8.8)	4 (3.1)
Insulin at Baseline *n* (%)	58 (66.7)	74 (77.1)	N/A	N/A
Ventilated at baseline, *n* (%)	58 (66.7)	70 (72.9)	95 (69.3)	93 (72.7)
APACHE Score, *n* (%) ***				
< 20	50 (57.5)	52 (54.2)	91 (66.4)	87 (68.0)
≥ 20	36 (41.4)	42 (43.8)	43 (31.4)	39 (30.5)
Unknown	1 (1.1)	2 (2.1)	3 (2.2)	2 (1.6)
CPIS score, n^a^/mean (SD) Baseline	56/9.6 (2.2)	63/9.5 (2.1)	89/9.5 (2.1)	91/9.2 (2.4)
Chest x-ray, *n* (%)^b^				
Unilateral	20 (23.0)	29 (30.2)	39 (28.5)	50 (39.1)
Bilateral	65 (74.7)	67 (69.8)	98 (71.5)	78 (60.9)
Pleural effusion, *n* (%)	42 (48.3)	38 (39.6)	63 (46.0)	58 (45.3)
Weight (kg), n^a^/mean (SD) ***	87/85.6 (26.8)	96/79.2 (22.4)	137/73.2 (18.0)	127/76.2 (20.4)
Comorbidities, *n* (%)				
Cardiac ***	58 (66.7)	69 (71.9)	73 (53.3)	68 (53.1)
Hepatobiliary	13 (14.9)	10 (10.4)	21 (15.3)	21 (16.4)
Gastrointestinal ***	57 (65.5)	56 (58.3)	71 (51.8)	63 (49.2)
Neoplastic	8 (9.2)	13 (13.5)	12 (8.8)	12 (9.4)
Renal/Urinary ***	40 (46.0)	53 (55.2)	43 (31.4)	41 (32.0)
Pulmonary	65 (74.7)	67 (69.8)	87 (63.5)	87 (68.0)
Vascular ***	38 (43.7)	37 (38.5)	38 (27.7)	41 (32.0)
Baseline pathogens, *n* (%)				
MRSA only	53 (60.9)	62 (64.6)	66 (48.2)	66 (51.6)
MRSA + other Gr (+)	6 (6.9)	9 (9.4)	16 (11.7)	9 (7.0)
MRSA + other Gr (-)	19 (21.8)	18 (18.8)	41 (29.9)	38 (29.7)
MRSA+ other Gr (+) and Gr (-)	5 (5.7)	3 (3.1)	8 (5.8)	10 (7.8)
MRSA + anaerobes	0	1 (1.0)	0	1 (0.8)
MRSA + fungal	4 (4.6)	3 (3.1)	6 (4.4)	4 (3.1)
Baseline Vancomycin MICs, *n* (%)				
≤ 0.5	7 (8.0)	2 (2.1)	10 (7.3)	12 (9.4)
1	63 (72.4)	75 (78.1)	105 (76.6)	93 (72.7)
2	7 (8.0)	7 (7.3)	6 (4.4)	11 (8.6)
4	0	1 (1.0)	0	1 (0.8)
unknown	10 (11.5)	11 (11.5)	16 (11.7)	11 (8.6)

*Abbreviations: CPIS* clinical pulmonary infection score, *MRSA* methicillin-resistant S. aureus, *SD* standard deviation

^a^Denotes the number of patients with available data when different than the group total

^b^Among diabetic patients treated with linezolid, 1 had a normal chest x-ray and one had unknown chest x-ray findings

*P < 0.05 for linezolid vs vancomycin treated diabetic patients; ** *P* < 0.05 for linezolid vs vancomycin treated non-diabetic patients; *** *P* < 0.05 for diabetic patients vs. non-diabetic patients

The baseline non-fasting blood glucose levels were similar between the diabetic and non-diabetic linezolid treated patients (mean ± SD 176.3 ± 116.7 mg/dL vs. 164.7 ± 152.7 mg/dL), and non-diabetic vancomycin treated patients (167.8 ± 123.5 mg/dL for diabetic vs. 174.9 ± 158.9 mg/dL) (Table 1).

### Treatment outcomes

**Fig. 1:**
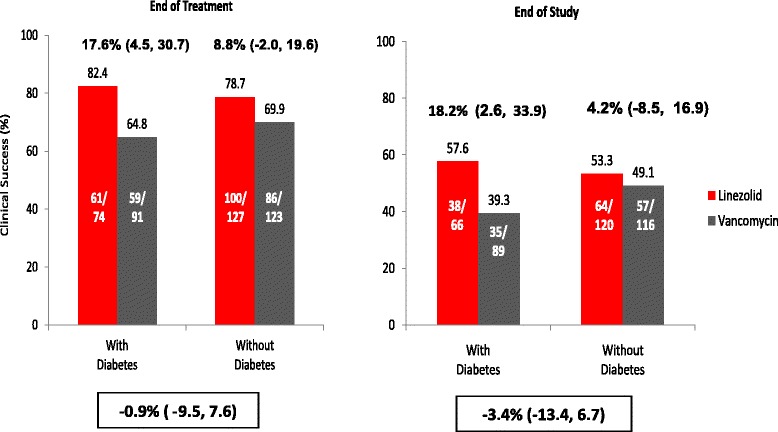
Clinical outcomes in patients with MRSA-NP treated with either linezolid or vancomycin by diabetes mellitus status in the MITT population. Risk Difference (RD) & 95 % Confidence Interval (CI) are presented for treatment group difference and for overall diabetes group differences in percent success. Percentages do not include patients with unknown outcomes

**Fig. 2:**
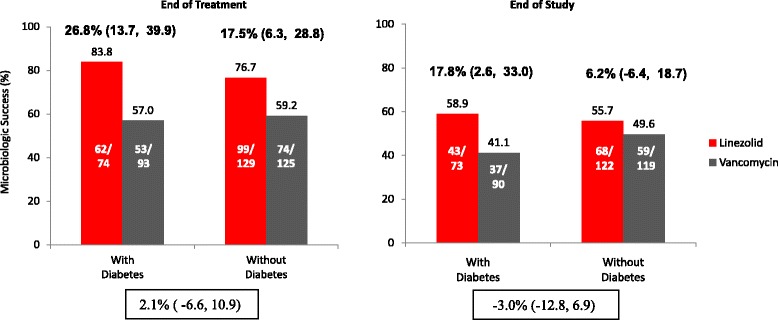
Microbiologic outcomes in patients with MRSA-NP treated with either linezolid or vancomycin by diabetes mellitus status in the MITT population. Risk Difference (RD) & 95 % Confidence Interval (CI) are presented for treatment group difference in percent success. Percentages do not include patients with unknown outcomes

The outcomes by diabetes subgroup are similar to those overall observed in the primary study, particularly in regards to EOS, EOT outcomes and mortality [12]. The duration of antibiotic therapy provided was similar for the two treatment arms and among the diabetic and non-diabetic patients (Table 1). On treatment days 3, 6 and 9 the vancomycin trough levels (in 03BCg/ml median [(min, max]) were 13.3 (4.4, 36.1), 15.0 (6.4, 41.4) and 18.0 (4.9, 26.2) among diabetic patients and 11.7 (2.8, 50.8), 14.7 (2.7, 45.0) and 13.7 (2.0, 46.9) among non-diabetic patients. The duration of ventilation and antibiotic treatment (data not shown), EOT and EOS overall clinical outcomes (Fig. 1) and microbiologic outcomes (Fig. 2) were similar between diabetic and non-diabetic groups.

Among the diabetic patients, clinical and microbiologic success rates at EOT and EOS were higher among linezolid treated patients as compared to those treated with vancomycin (Figs. 1 and 2). At EOT clinical success was noted in 82.4 % of diabetic patients treated with linezolid versus 64.8 % of diabetic patients treated with vancomycin (risk difference [RD], 95 % confidence intervals [CI]: 17.6 %, [4.5, 30.7]). Similarly, at EOT microbiologic success was noted for 83.8 % of diabetic patients treated with linezolid vs. 57.0 % of diabetic patients treated with vancomycin (RD, 95 % CI: 26.8 % [13.7, 39.9]). At the EOS visit, clinical success was found in 57.6 % of the diabetic patients treated with linezolid and 39.3 % of those treated with vancomycin [RD, 95 % CI: 18.2 % (2.6, 33.9)]; EOS microbiologic success was noted for 58.9 % of diabetic patients treated with linezolid and 41.1 % of those treated with vancomycin (RD, 95 % CI: 17.8 % [2.6, 33.0]).

Among the non-diabetic patients, clinical and microbiologic success rates at EOT and EOS were similar between treatment groups, except for clinical response at EOT (Figs. 1 and 2). At EOT, clinical success was noted in 76.7 % of non-diabetic patients treated with linezolid versus 59.2 % of non-diabetic patients treated with vancomycin (risk difference [RD], 95 % confidence intervals [CI]: 17.5 %, [6.3, 28.8]).

A multivariate regression analysis was conducted to evaluate the association between baseline factors and clinical response at EOT and EOS among patients with diabetes (Table 2). The final multivariate model for clinical response at EOT had the following predictors: combined MIC; vasopressor use; smoking status; study drug, bacteremia; and, pneumonia type. The final multivariate model for clinical response at EOS had the following predictors: race; and, vasopressor use. Diabetic patients treated with linezolid were 2.8 times more likely to have clinical success at EOT than those treated with vancomycin (*p* = 0.02). Second, diabetic patients without bacteremia accompanying their NP were 5 times more likely to have clinical success at EOT compared to those with bacteremia (*p* = 0.009). At EOS diabetic patients who were not on vasopressors at baseline were 3.3 times as likely to have clinical success compared to those who were, and Asian diabetic patients were almost 5 times more likely to have clinical success at EOS compared with white diabetic patients.

**Table 2:** Multivariate logistic regression modelling in patients with diabetes

Variable	Adjusted OR	95 % CI	*P* value
Clinical Success (mITT Population)			
End of Treatment			
Vasopressors at Baseline (Yes vs No)	0.4	(0.2, 1.1)	0.073
Treatment (LZD vs. VAN)	2.8	(1.2, 6.8)	0.022
Bacteremia (Yes vs No)	0.2	(0.1, 0.7)	0.009
Combined MIC (vs. 0.5)			
1	1.5	(0.2, 9.1)	0.673
2 or 4	0.4	(0.1, 3.2)	0.384
Pneumonia Type			
HCAP vs. HAP	2.9	(0.6, 13.7)	0.176
VAP vs. HAP	0.6	(0.2, 1.5)	0.239
HCAP vs. VAP	5.2	(1.3, 21.6)	0.023
Smoking Status			
Current vs. Non-Smoker	0.9	(0.3, 2.4)	0.774
Ex vs. Non-Smoker	2.3	(0.8, 6.1)	0.105
End of Study			
Vasopressors at Baseline	0.3	(0.1, 0.8)	0.019
Race (vs White)			
Asian	4.9	(1.7, 13.6)	0.002
Black	1.7	(0.7, 4.3)	0.281
Other	1.1	(0.2, 5.5)	0.885
Day 28 Mortality (mITT population)			
Age (10 years)	1.4	(1.1, 1.9)	0.014
Chronic Dialysis Care (Yes vs No)	5.9	(1.8, 19.6)	0.004
Race (vs. White)			
Asian	0.1	(0.0, 0.5)	0.004
Black	1.0	(0.4, 2.6)	0.987
Other	0.8	(0.1, 3.9)	0.735
Subject Type (vs. Medical)			
Surgical	1.0	(0.4, 2.2)	0.921
Trauma	1.3	(0.4, 5.1)	0.665
Bacteremia (Yes vs. No)	2.2	(0.8, 6.0)	0.115
Pleural Effusion (Yes vs. No)	0.5	(0.2, 1.0)	0.040

*CI* confidence interval, *mITT* modified intent-to-treat *OR* odds ratio

All-cause mortality within 28 days after randomization was higher among diabetic patients compared to non-diabetic patients (23.5 vs 14.7 %, RD = 8.8 %, 95 % CI [1.4, 16.3]), but was similar for linezolid and vancomycin treated diabetic patients (20.7 % for linezolid versus 26.0 % for vancomycin) and non-diabetic patients (17.5 % for linezolid versus 11.7 % for vancomycin). Results for all-cause mortality within 60 days after randomization were non-significantly higher among diabetic patients compared to non-diabetic patients [29.5 vs 23.0 %, RD = 6.5 %, 95 % CI (-1.8, 14.8)]. Adverse events resulting in death that were reported for more diabetic patients compared to non-diabetic patients were cardiovascular and cerebrovascular events, respiratory arrest and septic shock. Among diabetic patients, pneumonia, as an adverse event resulting in death, was reported for more linezolid-treated patients compared to vancomycin-treated patients. Adverse events resulting in death reported for more vancomycin-treated diabetic patients than linezolid-treated diabetic patients were multi-organ failure, respiratory failure, sepsis and septic shock.

A multivariate regression analysis was conducted to evaluate the association between baseline factors and day 28 mortality among patients with diabetes (Table 2). The final multivariate model for mortality had the following predictors: age; history of chronic renal dialysis; race; hospitalization service type; bacteremia; and, pleural effusion. There was a six-fold increased risk of death within 28 days of randomization among diabetic patients who were receiving chronic renal dialysis and a significantly increased risk of death with increasing age (a 1.4-fold increase for every 10 year increase in age). Furthermore, diabetic patients with pleural effusion were half as likely to die compared to diabetic patients without pleural effusion. Asian diabetic patients were 10 times less likely to die compared to white diabetic patients.

### Safety

Rates of treatment-related adverse events, as well as study drug discontinuations due to adverse events, were similar for diabetic and non-diabetic patients in the MITT population. The percentages of treatment-related adverse events, and study drug discontinuations were also similar between the two drug treatment groups (Table 3).

**Table 3:** Adverse events among diabetic and non-diabetic patients in the modified-intent-to-treat population

	Diabetic Patients (*N* = 183)		Non-diabetic Patients (*N* = 265)	
Adverse event (AE) n (%)	Linezolid *N* = 87	Vancomycin *N* = 96	Linezolid *N* = 137	Vancomycin *N* = 128
Patients with a treatment- related AE	22 (25.3)	26 (27.1)	46 (33.6)	43 (33.6)
Patients with a serious AE	33 (37.9)	45 (46.9)	56 (40.9)	36 (28.1)
Patients with study drug discontinuation due to AE	3 (3.4)	8 (8.3)	9 (6.6)	9 (7.0)
Patients with specific AEs:				
Diarrhea	12 (13.8)	14 (14.6)	28 (20.4)	20 (15.6)
Rash^a^	6 (6.9)	8 (8.3)	16 (11.7)	9 (7.0)
Nausea	7 (8.0)	7 (7.3)	10 (7.3)	11 (8.6)
Vomiting	3 (3.4)	4 (4.2)	8 (5.8)	8 (6.3)
Thrombocytopenia^d^	17 (20.7)	13 (14.3)	18 (13.5)	15 (12.3)
Kidney impairment^b^	3 (3.4)	13 (13.5)	5 (3.6)	9 (7.0)
IV site complications^c^	3 (3.4)	5 (5.2)	6 (4.4)	6 (4.7)

^a^Rash includes the following AE preferred terms: Genital rash, Rash, Rash erythematous, Rash generalized, Rash macular, Rash maculo-papular, Rash popular and Rash pustular

^b^Kidney Impairment includes the following AE preferred terms: Renal failure, Renal failure acute, Renal failure chronic and Renal impairment

^c^ IV Site Complications include the following AE preferred terms: Catheter site erythema, Catheter site haematoma, Catheter site haemorrhage, Catheter site infection, Catheter site pain, Infusion site cellulitis, Infusion site erythema, Infusion site extravasation, Infusion site infection, Infusion site phlebitis and Infusion site thrombosis

^d^Thrombocytopenia was defined as a platelet count of <150,000 μL, if platelet count was normal at baseline, or a 50 % drop if platelet count was not normal at baseline

## Discussion

Patients with diabetes mellitus have been reported to be at high risk for colonization and several types of infection with MRSA, especially pneumonia and soft tissue infections [13–17]. Furthermore, some previous studies suggest that diabetic patients with complicated MRSA skin and soft tissue infections respond less well to treatment compared to non-diabetic patients [18–20]. In a diabetic mouse model, treatment with either systemic linezolid or daptomycin had a more rapid therapeutic effect compared with vancomycin, but after day 1, all three antibiotics had similar efficacy against MRSA wound infection [21]. A study looking at the effect of the addition of a macrolide antibiotic to initial therapy with a third generation-beta lactam among patients with community acquired pneumonia did not show a difference between diabetic and non-diabetic subgroups [22].

We are not aware of any studies similar to ours on the role of co-morbid diabetes mellitus in patients with nosocomial pneumonia. A systematic review and meta-analysis published in 2013 found 9 studies comparing treatment of hospital-acquired pneumonia (of any cause) with linezolid versus vancomycin [23]. The results suggested that the two drugs had similar efficacy and safety profiles, including for infections caused by MRSA, but there was no breakdown in results based on the presence of diabetes. Thus, we conducted an analysis of data obtained from a recent large, randomized, prospective, multi-national clinical trial of culture confirmed MRSA NP. Our results show that treatment of diabetic patients with linezolid resulted in higher clinical and microbiologic success rates at EOT and EOS compared to treatment with vancomycin. In contrast, there were no significant differences for clinical and microbiologic success rates between the treatment arms among non-diabetic patients.

It would be reasonable to expect the random blood glucose levels to be higher in the diabetic patients, but they were not in this study. This may be because the diabetic patients had received prior treatment for glycemic control. In addition, the elevated blood glucose levels in the non-diabetic patients could at least be partly attributable to the stress of acute illness. It is also possible that some “non-diabetics” may be undiagnosed diabetics (or have pre-diabetes).

The concentration of vancomycin in lung epithelial lining fluid (ELF) is dependent upon blood vancomycin levels and alveolar capillary membrane protein permeability [18, 24], and is an important determinant of vancomycin antibacterial activity against MRSA in the lung [25]. Linezolid has been shown to have better ELF penetration than vancomycin in animal models of MRSA NP [26]. A meta-analysis of pulmonary function found that patients with diabetes have higher rates of restrictive lung disease [4], and postmortem histologic examination of lung tissue from patients with diabetes mellitus showed thickening of alveolar epithelial and pulmonary capillary basal laminae [27]. In our review of the literature we found no data on vancomycin ELF concentrations in patients with diabetes mellitus. In this double-blind clinical trial vancomycin trough levels were monitored and adjusted by local pharmacists and were similar between patients with and without diabetes mellitus; however, due to underlying lung pathology it may be that vancomycin penetration into pulmonary sites of infection is reduced, lessening the likelihood of cure of infection.

In our study the 28 day mortality rate for patients who had MRSA NP was higher among diabetic than non-diabetic patients, regardless of the treatment arm. A study by Falguera and colleagues reported an increased mortality among diabetic patients with community-acquired pneumonia that they attributed to underlying conditions, such as neoplastic disease, congestive heart failure, cerebrovascular disease, chronic renal disease, chronic obstructive pulmonary disease, chronic liver disease and HIV infection [28]. Patients with diabetes have also been shown to be at higher risk than non-diabetics for MRSA bacteremia [13, 29, 30]. In our study, the frequencies of multi-lobar pneumonia, pleural effusion and MRSA bacteremia were similar in the diabetic and non-diabetic patients with MRSA-NP. Perhaps not surprisingly, increasing age and undergoing chronic renal dialysis were associated with an increased mortality risk among diabetic patients. We also observed that the occurrence of cardiovascular and cerebrovascular events, as well as respiratory failure and septic shock, were events resulting in death more frequently reported for diabetic patients compared to non-diabetic patients. Of note, diabetic patients who were Asian, or who had pleural effusion at baseline were less likely to die from their MRSA-NP. Although recent studies suggest that body fat and lean mass content and fat distribution are different in Asians, and these differences may potentially lead to heterogeneity in the clinical presentation of age-related chronic diseases between ethnicities [31], we cannot assess the effect of race on treatment outcome from this study.

Our study has several limitations. First, it was based on a retrospective review of prospectively obtained data and was not planned a priori. Second, it is a subgroup analysis with a relatively small sample size in some groups, therefore making it difficult to identify small but potentially clinically important differences. Third, we conducted an interaction test that showed no significant differences in treatment responses between the diabetic and non-diabetic patients. Fourth, the diagnosis of diabetes in enrolled patients was based on information provided by the investigators. We had no data on the patient’s baseline hemoglobin A1C levels, which may have helped elucidate the relationship of prior glycemic control among patients with diabetes in the outcome of these infections. Additionally, diabetes mellitus is a disorder with a broad spectrum of severity. Identifying the type of diabetes (1 versus 2) and measurements of glycemic control (serum glucose or glycosylated hemoglobin) have limited ability to define the severity of the disease. As such, results should be interpreted with caution and considered as hypothesis-generating, rather than confirmatory. Given that treatment responses may differ between diabetic and non-diabetic patients, investigators in future clinical trials may wish to consider whether diabetic, pre-diabetic and non-diabetic groups should be stratified prior to randomization.

## Conclusions

A key finding in our study was that among diabetic patients with MRSA NP the cure rate was significantly higher among those treated with linezolid compared with those treated with vancomycin. Regardless of treatment arm, the 28-day mortality rate was higher among patients with diabetes than among non-diabetics, but were similar for linezolid and vancomycin treated diabetic patients. Increased mortality among the patients with diabetes mellitus was associated with increasing age and having received chronic renal dialysis. If our findings are replicated by other investigators, clinicians might consider treating diabetic patients with MRSA-NP with linezolid.

## Additional file

Additional file 1: Definitions of Clinical and Microbiologic Outcomes. Additional file 1: **Table S1.** defines the clinical outcomes [clinical cure, clinical improvement (only at EOT), clinical failure and indeterminate] and microbiologic outcomes [documented microbiologic eradication, presumed microbiologic eradication, presumed microbiologic persistence, superinfection, colonization and indeterminate]. (DOCX 14 kb)

## Abbreviations

AE: Adverse events
ELF: Epithelial lining fluid
EOS: End-of-study
EOT: End-of-treatment
ICUs: Intensive care units
IDSA: Infectious Diseases Society of America
MIC: Near zero variance, minimum inhibitory concentration
MITT: Modified-intent-to-treat population
MRSA: Methicillin resistant *Staphylococcus aureus*
NP: Nosocomial pneumonia
SAEs: Serious adverse events
