# Supplementary material for: The effect of diabetes mellitus on outcomes of patients with nosocomial pneumonia caused by methicillin-resistant Staphylococcus aureus: data from a prospective double-blind clinical trial comparing treatment with linezolid versus vancomycin
Source: BMC Infect Dis. 2016 Sep 6;16(1):476. doi: 10.1186/s12879-016-1779-5 (PMC5011934; doi:10.1186/s12879-016-1779-5)
Supplement: Additional file 1: — Definitions of Clinical and Microbiologic Outcomes. Additional file 1: Table S1. defines the clinical outcomes [clinical cure, clinical improvement (only at EOT), clinical failure and indeterminate] and microbiologic outcomes [documented microbiologic eradication, presumed microbiologic eradication, presumed microbiologic persistence, superinfection, colonization and indeterminate]. (DOCX 14 kb) [file 12879_2016_1779_MOESM1_ESM.docx]

**Additional file 1: Table S1: Definitions of Clinical and Microbiologic Outcomes**

| End point | Definition |
| --- | --- |
| **Clinical outcomes** |  |
| Clinical cure | Resolution of clinical signs and symptoms of pneumonia compared with baseline, improvement or lack of progression in chest imaging, and no requirement for additional antibacterial treatment |
| Clinical improvement  (used only at EOT evaluation) | Improvement in 2 or more clinical signs and symptoms of pneumonia compared with baseline, improvement or lack of progression of chest x-ray abnormalities, and no requirement for additional antibacterial treatment |
| Clinical failure | Persistence or progression of baseline signs and symptoms of pneumonia after at least 2 days of treatment, progression of baseline imaging abnormalities, or development of new pulmonary or extrapulmonary clinical findings consistent with active infection. Patients whose EOT assessment was a “failure,” but whose EOS assessment was missing, were considered failures. Patients who received any prohibited concomitant medication (ie, an antibiotic active against the patient’s MRSA isolate) were also assigned an EOS outcome of “failure” |
| Indeterminate | Extenuating circumstances preclude classification to 1 of the above |
| **Microbiologic outcomes** |  |
| Documented microbiologic eradication | Absence of MRSA from infection site |
| Presumed microbiologic eradication | Clinical cure without available microbiologic culture data |
| Presumed microbiologic persistence | Clinical failure in the absence of any microbiologic data |
| Documented microbiologic persistence | Continued presence of MRSA based on microbiologic culture |
| Superinfection | Clinical failure and isolation of a non-MRSA pathogen not present at baseline at the original infection site |
| Colonization | Clinical cure and isolation of a non-MRSA pathogen at the original site of infection |
| Indeterminate | Any patients who could not be classified into 1of the above categories |
